# Immune landscape and the key role of APOE+ monocytes of lupus nephritis under the single‐cell and spatial transcriptional vista

**DOI:** 10.1002/ctm2.1237

**Published:** 2023-04-07

**Authors:** Youzhou Tang, Ying Zhang, Xinyu Li, Ruoyao Xu, Ying Ji, Jishi Liu, Jianye Liu, Quan Zhuang, Hao Zhang

**Affiliations:** ^1^ Department of Nephropathy and Rheumatology The 3rd Xiangya Hospital Central South University Changsha China; ^2^ The Critical Kidney Disease Research Center of Central South University Changsha China; ^3^ Transplantation Center The 3rd Xiangya Hospital Central South University Changsha China; ^4^ Xiangya School of Medicine Central South University Changsha China; ^5^ Department of Urology The 3rd Xiangya Hospital Central South University Changsha China; ^6^ Research Center of National Health Ministry on Transplantation Medicine Changsha China

**Keywords:** APOE, immune landscape, lupus nephritis, single‐cell transcriptomic sequencing, spatial transcriptome

## Abstract

**Background:**

Lupus nephritis (LN) is among the most common complication of systemic lupus erythematosus (SLE) with high mortality and morbidity. The analysis of LN kidney's local immune response through single‐cell and spatial transcriptome enables the study of potential therapeutic targets.

**Methods:**

By single cell sequencing and spatial transcriptome, we profile cells from LN kidney and normal kidney tissues to characterize cellular composition and elucidate the potential upstream monocyte/macrophage (Mono/MΦ) initiating the auto‐immune response. After the high‐throughput synergy screening, we performed the immunofluorescence to identify the specific cells in LN patients. The function experiments were finished by flow cytometry and Elisa.

**Results:**

By immunofluorescence and spatial transcriptome, we identified differential subsets of Mono/MΦ and demonstrated that they exhibit temporal expression of TIMP1, IL1B, SPP1 and APOE. With the function experiments, we found that the APOE+ Mono may be compensatorily increased in LN, and the capacity of antigen presenting was decreased with the overexpression of APOE. Furthermore, how do the LN‐specific Mono/MΦ transport in and out the glomerulus to active the local immune response remains unclear. Our results showed that lymphangiogenesis occurred in LN kidneys but not in normal kidneys, suggesting the presence of a new lymphatic vessel may serve as a ‘green channel’ for LN‐specific Mono/MΦ.

**Conclusions:**

In LN, APOE+ Mono are compensatorily elevated, with decreased antigen presenting ability and reduced secretion of interferons. The lymphangiogenesis in LN prompts the trafficking of Mono/MΦ in LN kidney.

## Graphical abstract

Critical points: (1) Systemic lupus erythematosus is a self‐immune disease affecting multiple organs throughout the body, with lupus nephritis as the most common complication. (2) In lupus nephritis, APOE+ monocytes infiltrate the renal glomerulus and drain through newly formed lymphatic vessels. (3) Under stimulation of double‐stranded DNA, the antigen presenting function of APOE+ monocytes decreases and their ability to secrete IFNα and IFNγ is also reduced.

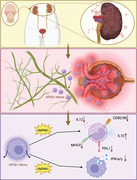

## 1 BACKGROUND

Systemic lupus erythematosus (SLE) is an autoimmune disease involving injuries of multiple systems and organs, among which lupus nephritis (LN) devotes the most to SLE mortality.^1^ Genetic background combined with environmental factors initiates LN pathogenesis, and the downstream immune network and signaling further accelerate LN progression to end‐stage renal disease. Immune cells and cytokines are the two main contents in LN immune network.^2^, ^3^ However, the detailed immune cells landscape of LN kidney, such as different kinds of myeloid cells and lymphocytes, have not been fully revealed in local lesion of LN patients.

In recent years, single‐cell RNA sequencing (scRNA‐seq) offers remarkable opportunities to systematically describe the cellular landscape of diverse diseases.^2^, ^4^ ScRNA‐seq can accurately measure mass genes inside each cell, thereby analyzing the heterogeneity of cells with the same phenotype between disease and normal status.^5^ Previous research has effectively utilized scRNA‐seq to create comprehensive immune cell profiles in the context of kidney disease.^6^, ^7^, ^8^, ^9^ Although scRNA‐seq has greatly expanded our understanding of the heterogeneity of immune cell subpopulations, performing scRNA‐seq requires dissociating cells from the tissue which results in a loss of information about the cellular spatial location and cell–cell interaction.^10^ Therefore, it is necessary to link gene expression with spatial location information. Additionally, integration of scRNA‐seq and spatial transcriptomics enables in situ visualization of signatures with mapping of a greater number of cell types than spatial transcriptomics alone.^11^, ^12^ Mapping these two kinds of mass data, we can determine the relative location of immune cells to parenchymal cells in disease, as well as specific molecular signatures within the lesion.^13^, ^14^

Monocytes, as a key component of the innate immune system, are critical for protecting the host against infectious agents and generating inflammation through the release of cytokines. Recent studies have revealed a link between monocyte activation and the progression of LN.^15^, ^16^ In human diffuse proliferative LN and in the nephritis model of lupus in MRL/LPR mice, the recruitment and activation of inflammatory cells are facilitated by monocyte chemoattractant protein (MCP)‐1+ monocytes.^17^ Depletion of colony stimulating factor‐1 (CSF‐1)+ monocyte decreased proteinuria, blood urea nitrogen (BUN) and improved renal histopathology in murine lupus model.^18^

Apoprotein E (APOE), a lipid‐transport protein, is almost secreted from liver, approximately 10% of which can be secreted by hepatic monocytes and kupffer cells.^19^ APOE is an endogenous immunomodulatory agent that affects both the innate and the adaptive immunity,^20^ involved in various diseases like Alzheimer's and cardiovascular diseases.^21^, ^22^ Furthermore, APOE+ Mono were proved to be potent antigen presenting cells (APCs) triggering CD8+ T cell activation in breast cancer.^23^ In our study, APOE+ Mono were analyzed to display the ability of down‐regulating antigen presentation in LN lesion and participate to inhibit pro‐inflammatory factors, such as interferons (IFNs), which might greatly contribute to the LN.

Another question worth exploring is how immune cells are trafficking in the local tissues. Over the past 10 years, remarkable progress has been achieved in the field of lymphatic vessels research, characterized by the identification of specific markers and the advancement of research tools. These advancements have greatly enhanced our comprehension of the role of lymphatic vessels in maintaining tissue homeostasis and the pathogenesis of diseases in various organs.^24^, ^25^ Lymphatic vessels possess diverse immunoregulatory capabilities through their expression of an extensive array of chemokines and receptors. This highlights that lymphatic vessels are not just channels for the removal of lymph, immune cells and cellular products from tissues and organs but have much more to offer in terms of their immunoregulatory functions.^26^, ^27^ The study of draining lymphoid systems in kidney has also caused for researchers’ concern. Unlike resident immune cells, a considerable part of immune cells especially myeloid cells infiltrates to local LN inflammatory environment to exert their effect through draining lymphoid systems.^28^, ^29^ Therefore, the origin and concrete trafficking pathway of these cells are another important mechanism in LN. In the kidney, most lymphatic vessels exist in the cortex.^30^ By the pressure differential of hydrostatic pressure and osmotic pressure, lymphatic vessels drain excess tissue fluid from surrounding tissues to regulate the homeostasis and send tissue fluid to lymphoid tissues or organs for facilitating the trafficking and activation of immune cells.^31^ There was only a few research of lymphatic system in kidney diseases, including acute kidney injury,^32^, ^33^ polycystic kidney disease^34^ and hypertensive nephropathy.^35^ Nevertheless, owning the difficulty in visualizing the typically small and sparse lymphatic vessels, and the complicated immune process, the research of lymphangiogenesis and lymph node in LN has been overlooked which we infer to play important role in immune cell transportation.

Here, we present an integration of scRNA‐seq and spatial transcriptomics to study the immune landscape of LN. Specific immune‐cell subsets of both normal and LN kidneys were identified and mapped onto the spatial transcriptome slices. APOE+ Mono were shown as the beneficial monocyte/macrophage (Mono/MΦ) in the upstream of LN immune response. Moreover, our study identified lymphangiogenesis in LN through immunofluorescence analysis, sorted out the key monocyte subcluster involved in regulating the inflammatory process, and explored the trafficking mechanism of specific immune cell subsets in the pathogenesis of LN.

## 2 METHODS

### 2.1 Collection of LN and normal renal samples

Renal tissue from patients with LN (3 IV‐G(A/C) and 1 IV‐G(A/C)+V) was acquired from the 3rd Xiangya Hospital, Central South University under a normative renal biopsy procedure. Control kidney samples were obtained from kidney surgery of non‐autoimmune renal disease patients. The study was reviewed and approved by the Institutional Review Board (Ethics Committee) of the 3rd Xiangya Hospital, Central South University (number: 21029).

### 2.2 Single cell preparation

Within 30 min of procurement from both LN and normal donors, the renal tissue samples were preserved in the sCelLiveTM Tissue Preservation Solution (Singleron, China) on ice. The samples were then rinsed three times with Hanks Balanced Salt Solution, cut into small pieces, and digested using 3 mL of sCelLiveTM Tissue Dissociation Solution with the Singleron PythoN Tissue Dissociation System at 37°C for 15 min. The cell mixture was gathered and passed through a 40‐micron sterile filter to separate the cells. After that, the GEXSCOPE red blood cell lysis buffer (RCLB, Singleron) was added to the mixture in a 1:2 (volume) ratio with the cells and incubated at room temperature for 5–8 min to eliminate red blood cells. The mixture was then centrifuged at 300xg and 4°C for 5 min to remove the supernatant and resuspended softly in phosphate buffered saline (PBS) (HyClone, USA).

### 2.3 Single‐cell RNA sequencing

The procedure involved loading 2 × 10^5^ cells/mL in PBS as single‐cell suspensions onto a microwell chip using the Singleron Matrix Single Cell Processing System. Then, barcoding beads were collected from the chip, followed by reverse transcription of the captured mRNA to generate cDNA. After that, polymerase chain reaction (PCR) amplification was performed on the cDNA. The amplified cDNA was fragmented and ligated with sequencing adapters to construct scRNA‐seq libraries using the protocol from the GEXSCOPE Single Cell RNA Library Kits (Singleron).^36^ Finally, the individual libraries were diluted to 4 nM, combined into a pool, and sequenced using the Illumina Novaseq 6000 platform with 150 bp paired‐end reads.

### 2.4 Quality control, dimension‐reduction and clustering of scRNA‐seq and spatial transcriptomics

Cells were filtered based on gene counts between 0 and 5500 and unique molecular identifer (UMI counts lower than 70 000. Cells with over 95% mitochondrial content were discarded (Figure S1A–C). We utilized functions from Seurat v4.1.1^37^ for dimension reduction and clustering. Next, we normalized and scaled all gene expression using the NormalizeData and ScaleData functions and selected the top 4000 variable genes for principal component analysis (PCA) analysis using the FindVariableFeatures function (Figure S1D). Using the top 30 principal components, we grouped cells into multiple clusters using the FindClusters function. Batch effects between samples were corrected with the Harmonization method. Lastly, the uniform manifold approximation and projection (UMAP) algorithm was employed to visualize the cells in a two‐dimensional space.

### 2.5 Spatial transcriptomics

Tissues were frozen in isopentane (Sigma‐Aldrich, USA) and then embedded in optimal cutting temperature compound (OCT). The embedded tissue blocks were cryosectioned in a cryostat to generate appropriately sized sections for Visium Spatial slides while keeping the samples frozen. Sections were placed respectively on Visium Spatial Tissue Optimization Slide and Visium Spatial Slide within the capture area. H&E staining and microscope brightfield imaging for sections were then processed. Sample fixing and imaging have been done in sample preparing, and section permeabilization will be performed as follows. Permeabilization processes for the time determined by tissue optimization. The first strand of cDNA was synthesized via reverse transcription and the second strand of cDNA was synthesized via PCR. Then the cDNA was denaturation, making the second strand of cDNA dissociated from slide. The full‐length cDNA, which was spatially barcoded, was amplified by PCR to produce enough mass for library construction. The process involved enzymatic fragmentation and size selection to improve the size of the cDNA amplicon. The P5, P7, i7 and i5 sample indexes, as well as the TruSeq Read 2 primer sequence, were added through End Repair, A‐tailing, Adaptor Ligation and PCR processes. The resulting libraries included the P5 and P7 primers were used for Illumina amplification.

### 2.6 Identification of clusters in spatial transcriptomics

We used functions from Seurat v4.1.1^37^ or dimension‐reduction and clustering. The function of SCTransform was used to normalize and scale all gene expression. After dimensionality reduction and clustering of RNA expression data, UMAP algorithm was applied to visualize cells in a two‐dimensional space.

### 2.7 Differential‐expression analysis

To determine which genes were differentially expressed, we utilized the Seurat ‘FindMarkers’ function and conducted a Wilcox likelihood‐ratio test. The genes considered were those expressed in at least 10% of cells within a cluster and showed an average log fold change of greater than .25. To classify the cell types in each cluster, we analyzed the expression of canonical markers, referenced literature, and created heatmaps, dot plots, and violin plots using Seurat's ‘DoHeatmap’, ‘DotPlot’ and ‘Vlnplot’ functions. Finally, any cells displaying markers for different cell types were manually removed as doublets.

### 2.8 Pathway enrichment analysis

To identify the potential roles of DEGs, we performed gene ontology (GO) and Kyoto Encyclopedia of Genes and Genomes (KEGG) analysis using the ‘clusterProfiler’ R package version 3.16.1. The pathways that showed a p‐value adjusted to be less than .05 were considered to have significant enrichment. The GO gene sets, encompassing molecular function, biological process and cellular component categories, were utilized as the reference. For gene set variation analysis (GSVA) pathway enrichment analysis, we utilized the average gene expression of each cell type as the input data with the help of the GSVA package.

### 2.9 Calculation of gene set‐based IFN scores

The function of AddModuleScore in ‘Seurat’ package was used to analysis the IFN‐related genes based on the average expression level.

### 2.10 Cell‐cell communications with NicheNet

To examine the relationship between immune cells, NicheNet was utilized. The analysis focused on ligand and receptor interactions, where genes expressed in at least 10% of cells in the clusters were considered. The top 100 ligands and 1000 targets of DEGs among ‘sender cells’ and ‘affected cells’ were selected for the paired ligand‐receptor analysis. A heatmap was produced using Nichenet_output$ligand_activity_target_heatmap to display the regulatory activity of ligands. The scores ranged from 0 to 1. Additionally, the average expression of ligands and receptors was presented in a heatmap by calculating the average gene expression within specific cell types and normalizing it across subtypes.

### 2.11 Trajectory reconstruction

We reconstructed the cell differentiation pathway using Monocle3 by sorting the cells based on the differential gene expression. We utilized the UMAP method for feature selection and dimension reduction and visualized the trajectory using the plot_cells function.

### 2.12 Immunofluorescence

Frozen section pieces were dried under room temperature and fixed by aceton for 10 min. Samples were then washed by .01 M PBS and applied on 1.2% H_2_O_2_ for 30 min to exclude nonsopecific staining. After three times wash by PBS and one time by .3% Triton X‐100, primary antibodies diluted in suitable concentrations (APOE (abcam, ab183597, rabbit)+CD14 (PTG, 60253‐1‐Ig, mouse), IFN‐a (PTG, 18013‐1‐AP, rabbit)+CD14, IL‐1B (PTG, 16806‐1‐AP, rabbit)+CD14, LYZ (PTG, 15013‐1‐Ap, rabbit)+CD14, PDPN (PTG, 11629‐1‐AP, rabbit)+VEGFC (Santa, Sc‐273628, mouse), SPP1 (abcam, ab63856, rabbit)+CD14) and VEGFC+CD14 were incubated with samples under 4°C overnight. After three times of .01 M PBS washes, secondary antibodies were incubated for 2 h. Section samples were then modulated on mounting medium and observed by fluorescence microscope (40 × 10, 20 × 10).

### 2.13 Cell culture and transfection

Mouse monocytic cell line (RAW264.7) was obtained from the cell bank of Chinese Academy of Sciences (Shanghai, China). RAW264.7 monocytes were cultured in DMEM medium (HyClone, USA), supplemented with 10% fetal bovine serum (FBS) and 1% penicillin–streptomycin at 37°C and 5% CO_2_. Logarithmically growing RAW264.7 monocytes were subjected to different treatments as follows: (1) Blank control: RAW264.7 monocytes were left untreated. (2) HSV: RAW264.7 monocytes were transfected with dsDNA plasmid, (3) HSV+Empty Vector: RAW264.7 monocytes were co‐transfected with dsDNA plasmid and empty vector plasmid. (4) HSV+si‐APOE: RAW264.7 monocytes were transfected with APOE siRNA plasmid. (5) HSV+oe‐APOE: RAW264.7 monocytes were transfected with APOE overexpression plasmid. Remove dsDNA plasmids, no‐load plasmids, APOE overexpression plasmids and APOE siRNA plasmids and thaw on ice. Add 95 uL of serum‐free DMEM medium to the centrifuge tube, then add 3 ug of APOE overexpression plasmid or 5 ul of APOE siRNA plasmid and 5 uL of Lip2000 to the centrifuge tube, respectively. Others are also added to the corresponding centrifuge tubes according to this method. Gently mix and allow to rest for 25 min at room temperature. Finally, the mixture is evenly added to the wells to be transfected and mixed. After incubation in a 37°C incubator for 6 h, replace it with fresh complete culture medium for inspection.

### 2.14 Enzyme‐linked immunosorbent assay (ELISA)

According to the manufacturer's instructions, the concentration of IFN‐α and IFN‐γ in the culture supernatants was detected by ELISA kit.

### 2.15 Flow cytometry

After detaching the RAW264.7 cellsfrom the bottom of 6‐well cell culture plates. For surface staining, cells were washed by the pre‐chilled PBS (pH 7.2–7.4), followed by suspension within PBS to 1 × 10^6^ cells/ml. Briefly, the 5‐ml tube was added with 100‐µL cell suspension, followed by staining with CD80‐BV605 (2 µL, Cat: 563052, BD, USA), CD86‐PE‐Cy7 (2 µL, Cat: 560582, BD, USA), MHCII‐Alexa Fluor 700 (2 uL, Cat:107622,biolegend,USA), PDL1‐BV421(2 uL, Cat:564716, BD, USA). For intracellular staining, cells were stimulated for 4 h with 1× Cell Stimulation Cocktail (Cat: 00‐4970‐93, eBioscience,USA) and 1× Protein Transport Inhibitor Cocktail (Cat: 00‐4980‐03 eBioscience,USA) according to the manufacturer's instructions. After incubation, cells were stained with IL10‐APC(3 uL, Cat:554468, BD, USA),IL12‐PE (3 uL, Cat: 554479, BD, USA). Flow cytometry was performed using a BD FACS Canto II after 25 min of incubation, and the result were analyzed using FlowJo 10.4 software (Tree Star, Ashland, OR, USA)

### 2.16 Statistical analysis

Data were represented as mean ± standard deviation (SD). Significant differences between control and experimental groups were compared through one‐way ANOVA and multiple unpaired t test by using SPSS17.0. A difference of *p* < .05 indicated statistical significance.

## 3 RESULTS

### 3.1 A single‐cell transcriptomic atlas of paired human LN and normal renal tissues

**FIGURE 1:**
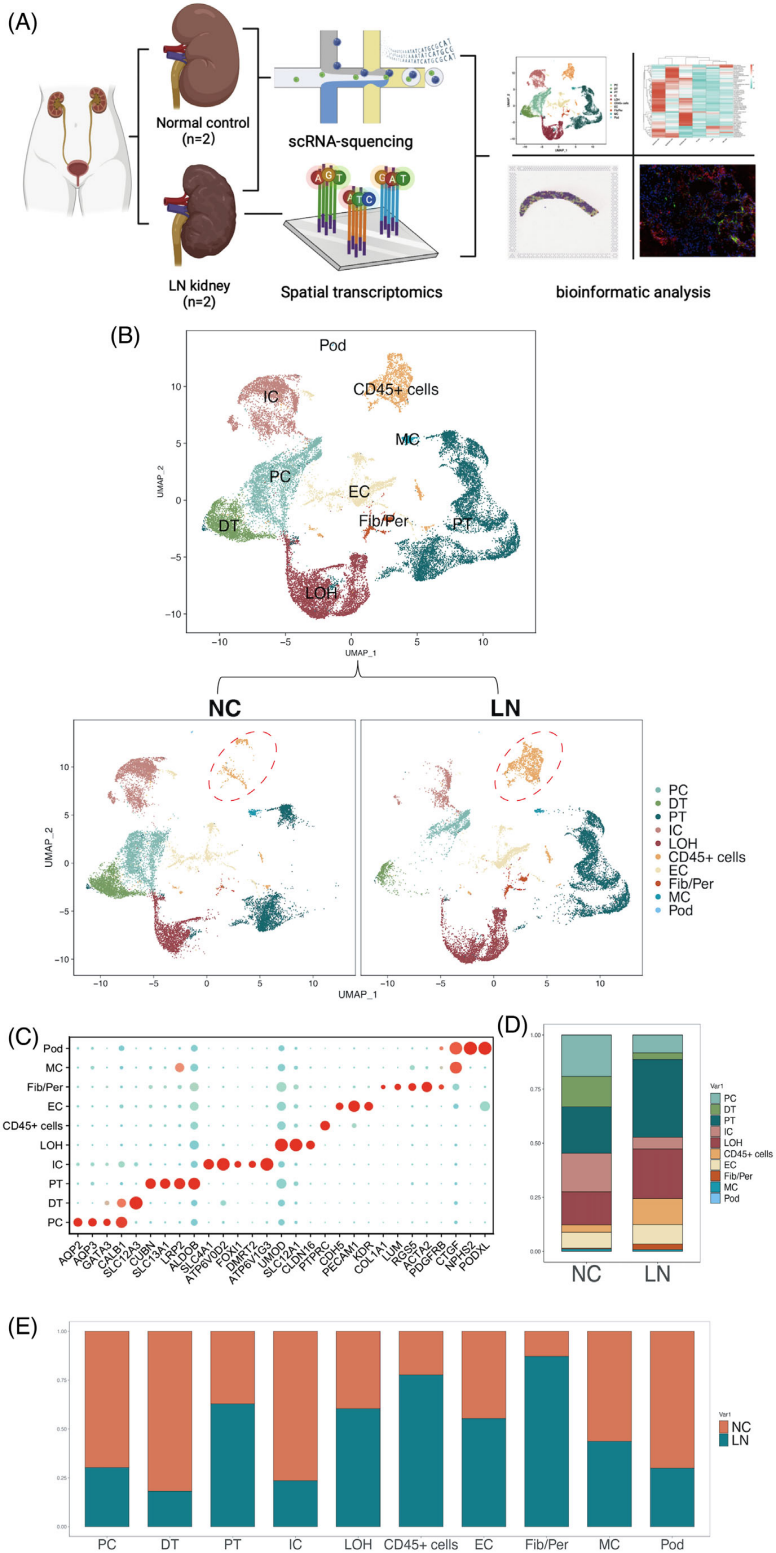
Single‐cell atlas of paired human normal kidney and lupus nephritis (LN) kidney tissues. (A) Graphic overview of this study design. Normal kidney and LN kidney tissues were used for scRNA‐seq. The other LN kidney tissues from two LN patients were used for spatial transcriptomics. (B) Uniform manifold approximation and projection (UMAP) plots of 15.773 cells from normal kidney and 15 965 cells from LN kidney tissue, showing 10 clusters in each plot. Each cluster was shown in different color. (C) Dot plots showing average expression of known markers in indicated cell clusters. The dot size represents percent of cells expressing the genes in each cluster. The expression intensity of markers is related to the color. (D) Ratio of 10 major cell types showing in bar plots in different group (in NC group, principal cell [PC]: 19.18%, distal tubule cells [DT]: 13.99%, PT: 21.46%, intercalated cell [IC]: 17.85%, loop of henle cells [LOH]: 15.17%, CD45+ cells: 3.46%, endothelial cell [EC]: 7.38%, Fib/Per: .39%, mesangial cell [MC]: .99%, Pod: .13%; in LN group, PC: 8.24%, DT: 3.07%, PT: 35.93%, IC: 5.44%, LOH: 22.92%, CD45+ cells: 11.94%, EC: 9.04%, Fib/Per: 2.60%, MC: .76%, Pod: .06%). (E) Ratio of 10 major cell types showing in bar plots in different group (NC versus LN, PC: 69% vs. 31%, DT: 82% vs. 18%, PT: 37% vs. 63%, IC: 76% vs. 24%, LOH: 40% vs. 60%, CD45+ cells: 22% vs. 78%, EC: 45% vs. 55%, Fib/Per: 13% vs. 87%, MC: 56% vs. 44%, Pod: 70% vs. 30%).

To elucidate the cellular composition of LN, kidney tissue was collected from LN patients (*n* = 2) and comparably normal renal tissue after nephrectomy of kidney tumour patients (*n* = 2) as normal control (NC), the basic information of samples was presented in the Table S1. The specimens were immediately processed to 3′‐end scRNA‐seq (Figure 1A). After removing damaged or dead cells and potential duplicates from the scRNA‐seq data, 31 738 cell transcriptomes were retained for further analysis. Following the removal of damaged or dead cells and putative cell doublets, a total of 31 738 cell transcriptomes were retained for analysis from the four patients in the scRNA‐seq data. The gene expression was normalized to account for sequencing depth and mitochondrial read count and PCA was then performed based on the highly variable genes across the cells. The cells were annotated using lineage markers (Figure 1B), which were later confirmed with published gene signatures..^38^, ^39^, ^40^ The cells were classified into 10 major cell types (Figure 1B–D) from 25 subsets(Figure S1E) including principal cells (PC; *n* = 4342) classified through the presence of certain markers *AQP2*, *AQP3* and *GATA3*, distal tubule cells (DT; *n* = 2697) which expressed *CALB1* and *SLC12A3*, proximal tubule cells (PT; *n* = 9120) marked by *CUBN*, *SLC13A1*, *LRP2* and *ALDOB*, intercalated cells (IC; *n* = 3685) identified by *SLC4A1*, *ATP6V0D2*, *FOXI1*, *DMRT2* and *ATP6V1G3* expression, loop of henle cells (LOH; *n* = 6051), which expressed *UMOD*, *SLC12A1* and *CLDN16*, CD45+ cells (*n* = 2452), identified by *PTPRC*, endothelial cells (EC; *n* = 2608), which were identifiable by specific marker gene *CDH5*, *PECAM1* and *KDR*, fibroblasts/pericytes (Fib/Per; *n* = 476), which are mixed marked by *COL1A1*, *LUM*, *RGS5*, *ACTA2* and *PDGFRB*, mesangial cells (MC; *n* = 277) marked by *CTGF*, and podocytes (Pod; *n* = 30) marked by *NPHS2* and *PODXL* (Figure 1C). Our findings demonstrated that while the 10 primary cell types were present in both the LN and normal renal tissue samples of the four patients, the extent of infiltration varied among these cell types, and the CD45+ cells were markedly elevated in LN patients (Figure 1D,E, Figure S1F), reflecting the immune cells involved in the LN.

### 3.2 Spatial transcriptomic cell‐type localization in LN renal tissues

**FIGURE 2:**
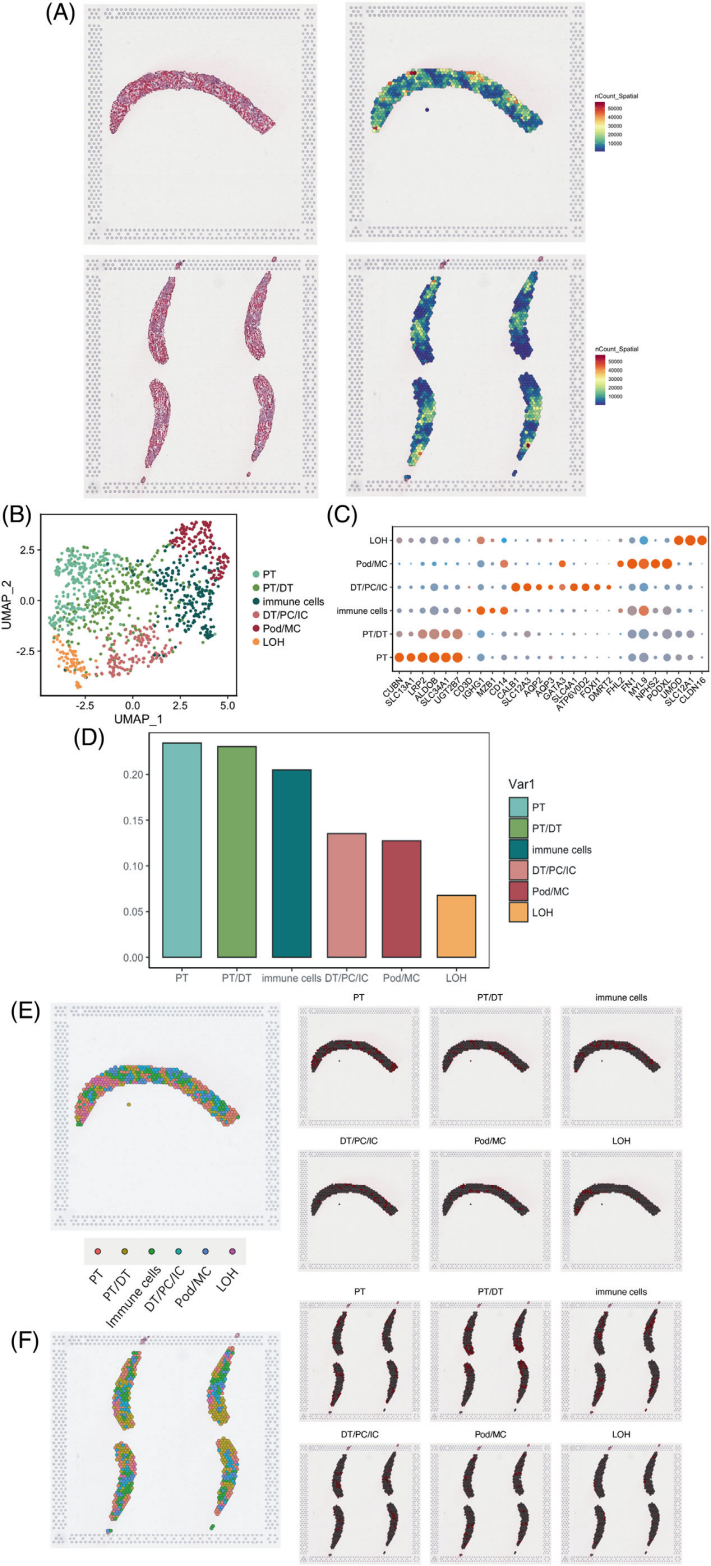
Spatial transcriptome atlas of paired human lupus nephritis (LN) kidney tissues. (A) The hemetoxylin and eosin (H&E) stain and UMI counts of LN kidney tissues. (B) Uniform manifold approximation and projection (UMAP) plots of two merged spatial transcriptome of LN kidney tissue, showing six clusters in each plot. Each cluster was shown in different color. (C) Dot plots showing average expression of known markers in indicated cell clusters. The dot size represents percent of cells expressing the genes in each cluster. The expression intensity of markers is shown. (D) Ratio of six major cell types showing in bar plots (PT: 23.43%, PT/distal tubule cells [DT]: 23.04%, immune cells: 20.49%, DT/principal cell [PC]/intercalated cell [IC]: 13.53%, Pod/mesangial cell [MC]: 12.75%, loop of henle cells [LOH]: 6.76%). (E and F) The spatial distribution of six cell types in the LN kidney tissues.

Our aim was to associate transcriptomic signatures with specific histological structures on a section, we collected LN renal tissues from two LN patients. After applied SCTransform to normalize the different sequencing depth, the SpatialFeaturePlot of gene count in the slice showed that all gene expression levels in the vicinity of the glomerulus were higher than that in the interstitium (Figure 2A), which may indicate that a variety of nucleated cells, including immune cells, were chemotactically infiltrated around the glomerulus, thereby promoting the progression of inflammation. The outcome of the spatial transcriptomic mapping was a collection of small circular areas, each measuring 55 µm in diameter and having a distinct gene expression pattern. Note that 1020 spots and 53 609 features were measured in these two slices. Our study used the UMAP method to determine and distinguish six mixed cell clusters based on expression of established markers (Figure 2B,C). The immune cells were identified by the multiple immune‐cell markers and the low expression of renal cell markers. High percentage of immune cells was observed in the sections (Figure 2D). The expression patterns of PT, DT, immune cells, DT/PT/IC, Pod/MC and LOH can be visualized. The transcriptomic spots that are associated with genes related to glomerular structures were clearly identified and located over the histologically identified glomeruli. Immune cells were distributed surrounding inside and outside the glomerulus (Figure 2E,F). These results demonstrate that immune infiltration of the glomeruli may be associated with LN.

### 3.3 Immune‐cell subsets analysis in LN and normal renal tissues

**FIGURE 3:**
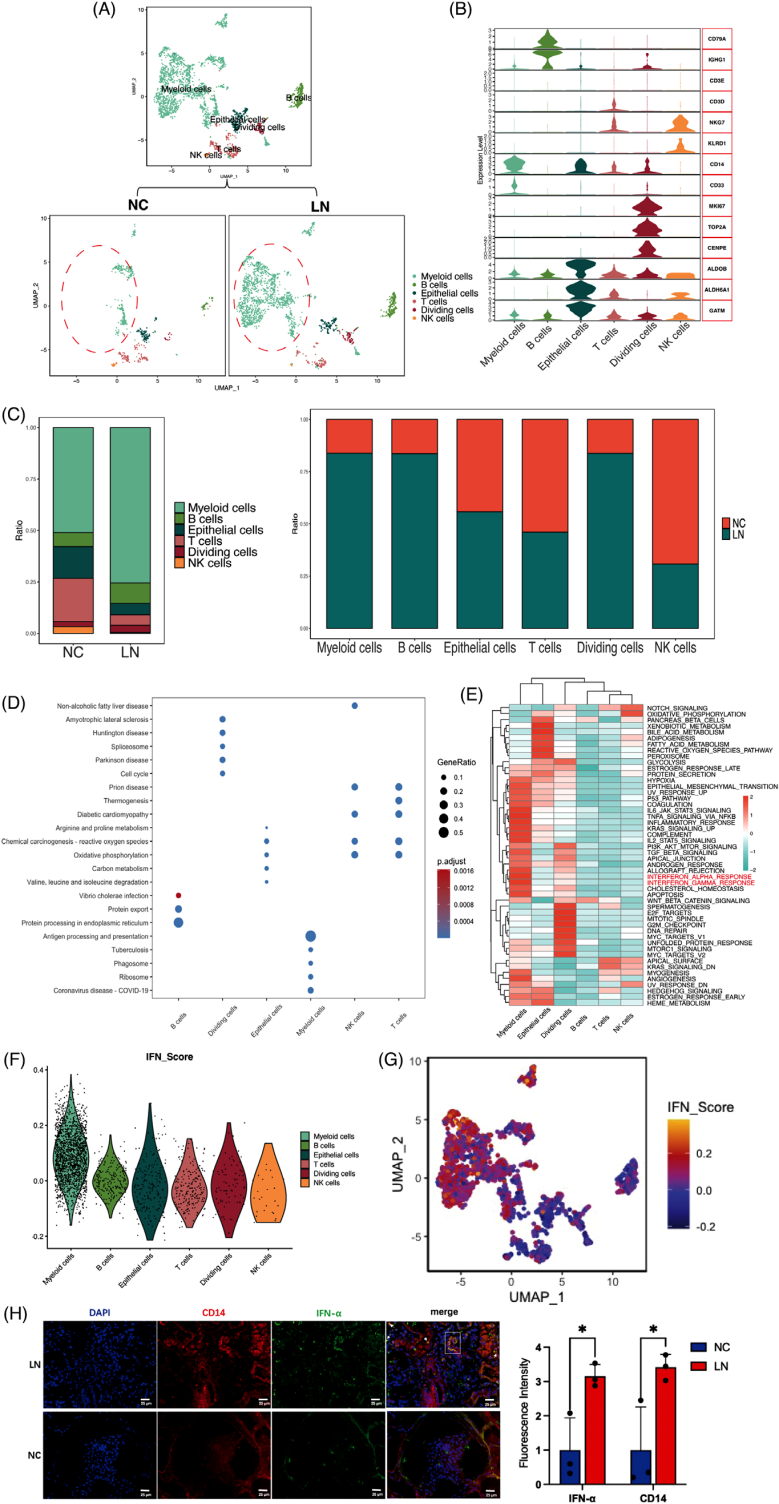
Characterization of immune cells in human normal kidney and lupus nephritis (LN) kidney tissues. (A) Uniform manifold approximation and projection (UMAP) plots showing the composition of CD45+ immune cells colored by cluster. (B) Violin plots showing the expression of selected genes. (C) Ratio of six major cell types showing in bar plots in different group (left panel, in NC group, myeloid cells: 51.01%, B cells: 6.79%, epithelial cells: 15.41%, T cells: 21.10%, dividing cells: 2.39%, NK cells: 3.30%; in LN group, myeloid cells: 75.46%, B cells: 9.91%, epithelial cells: 5.56%, T cells: 5.14%, dividing cells: 3.51%, NK cells: .42%), tissues (right panel, NC versus LN, myeloid cells: 16% vs. 84%, B cells: 16% vs. 84%, epithelial cells: 44% vs. 56%, T cells: 54% vs. 46%, dividing cells: 16% vs. 84%, NK cells: 69% vs. 31%) are shown. (D) Kyoto Encyclopedia of Genes and Genomes (KEGG) enrichment showing the related functions of immune cells. (E) GSVA enrichment showing the hallmarks for differentially expressed genes in the different immune cell types. (F) Violin plot showing the distribution of the interferons (IFN) response score in immune cells. (G) UMAP projection of IFN response score in immune cells. (H) Double immunofluorescence staining of CD14 and IFN‐α in human normal kidney and LN kidney tissues, data are presented as means ± SD and distributions compared by multiple unpaired *t* test (*p* < .05*; *p* < .01**; *p* < .001***; *p* < .0001****).

Analysis of CD45+ cell subsets from all renal cells in two groups with UMAP projections revealed six subclusters including myeloid cell, B cells, epithelial cells, T cells, dividing cells, natural killer (NK) cells (Figure 3A). We analyzed the expression of immune cell markers *CD79A*, *CD3D*, *CD14*, *CD33*. And the markers represent cell division and proliferation including *MKI67*, *TOP2A* and *CENPE* (Figure 3B). Among these immune‐cell subclusters, myeloid cells are the capital cluster in absolute numbers (Figure 3C). Compared to normal tissues, the number of myeloid cells was significantly elevated. KEGG analysis showed that highly expressed genes in myeloid cells were most enriched in antigen processing and presentation, suggesting that the antigen processing took great part in the LN pathogenesis. The genes related to oxidative phosphorylation were enriched in NK and T cells, indicating that the metabolic state of immune cells may also change (Figure 3D). To further investigate the pathways networks of immune‐cell subsets, we analyzed the changes in pathways of various immune cells, including B cells, T cells, myeloid cells, dividing cells, epithelial cells and NK cells, by utilizing the hallmark gene sets of the Molecular Signatures Database (MsigDB)^41^ (Figure 3E). The enrichment results showed that the IFN‐α and IFN‐γ responses were most enriched in myeloid cells, suggesting myeloid cells participated in the autoimmune response of LN through the secretion and response of IFN. To deeply exploring this hypothesis, we calculated the IFN score by the function of AddModuleScore in ‘Seurat’ package. We found that the enrichment score of the myeloid cells was relatively higher than others (Figure 3F,G). Meanwhile, immunofluorescent staining showed the consistent results (Figure 3H). Together, our results indicated that myeloid cells may play an important role in IFN response of LN. Moreover, we also analyzed B cells and T cells, and the specific details were presented in the Figures S2 and S3.

### 3.4 Specific dendritic cells subsets in LN

**FIGURE 4:**
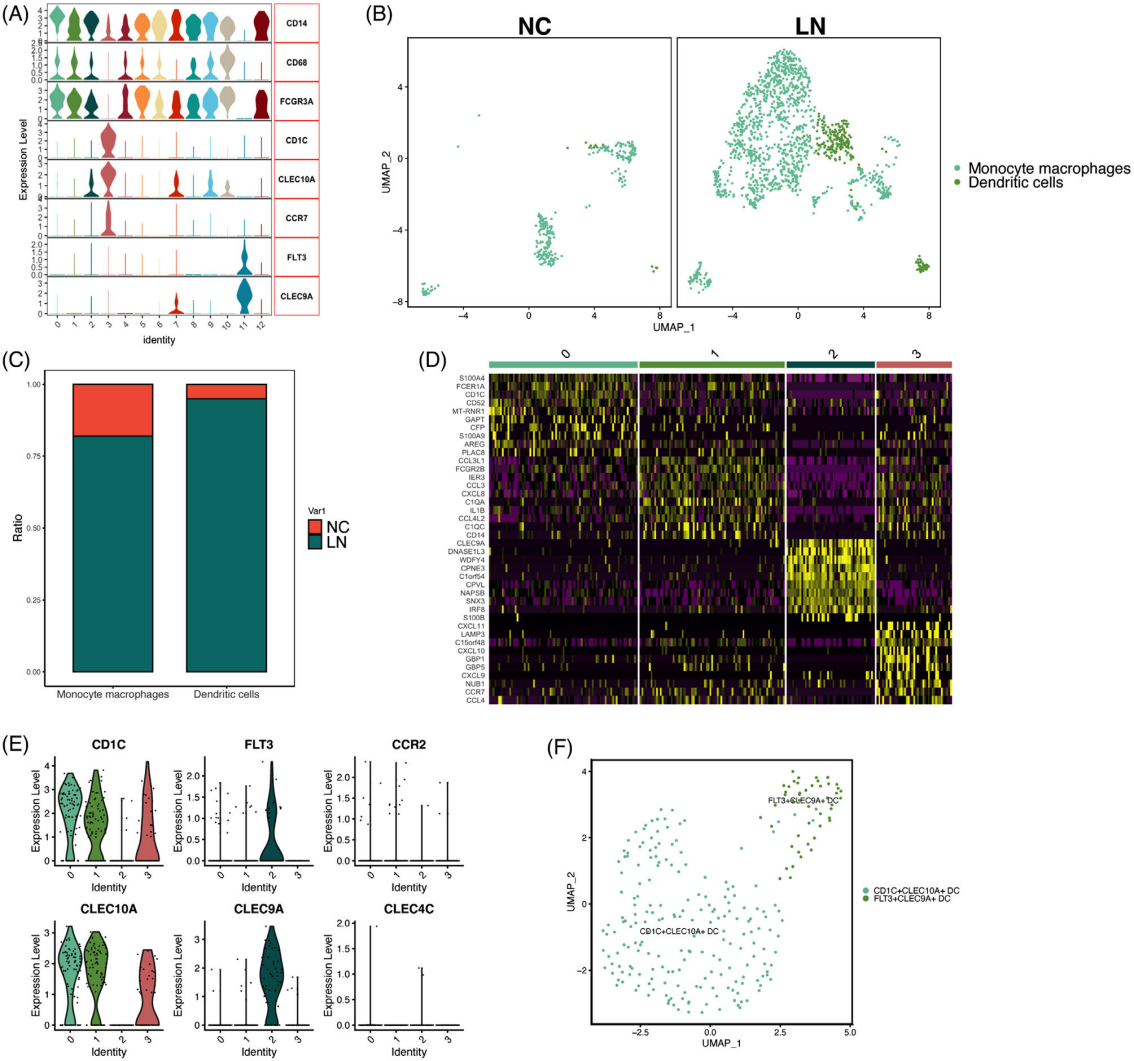
Characterization of myeloid cells and dendritic cells (DC) in human normal kidney and lupus nephritis (LN) kidney tissues. (A) Violin plots showing the expression of selected genes. (B) Uniform manifold approximation and projection (UMAP) plots showing the composition of myeloid cells colored by cluster. (C) Ratio of two major cell types showing in bar plots (NC versus LN, Monocyte macrophages: 18% vs. 82%, dendritic cells: 5% vs. 95%). (D) Heatmap showing the top 10 genes in each cluster of DC. (E) Violin plots showing the marker genes of DC. (F) UMAP plots showing the composition of DC colored by cluster.

When it comes to myeloid cells, the classical markers of dendritic cells (DCs) and Mono/MΦ were used to identify the subclusters (Figure 4A). Finally, analysis of myeloid cells between two groups with UMAP projections revealed two subclusters: DC and Mono/MΦ (Figure 4B). Mono/MΦ almost accounted for 80% of myeloid cells in LN patients (Figure 4C). However, in normal kidneys, DCs were practically absent (Figure 4C). The top DEGs of these subclusters were shown in heatmaps (Figure 4D), a large part of which were the genes of the chemokine family. In DC subclusters, *CD1C* and *CLEC10A* were highly expressed in clusters 0, 1 and 3. Cluster 2 expressed *FLT3* and *CLEC9A* (Figure 4D‐E). In the UMAP projections, DCs were clearly separated into CD1C+CLEC10A+ and FLT3+CLEC9A+ DC.

### 3.5 Specific Mono/MΦ subsets in LN

**FIGURE 5:**
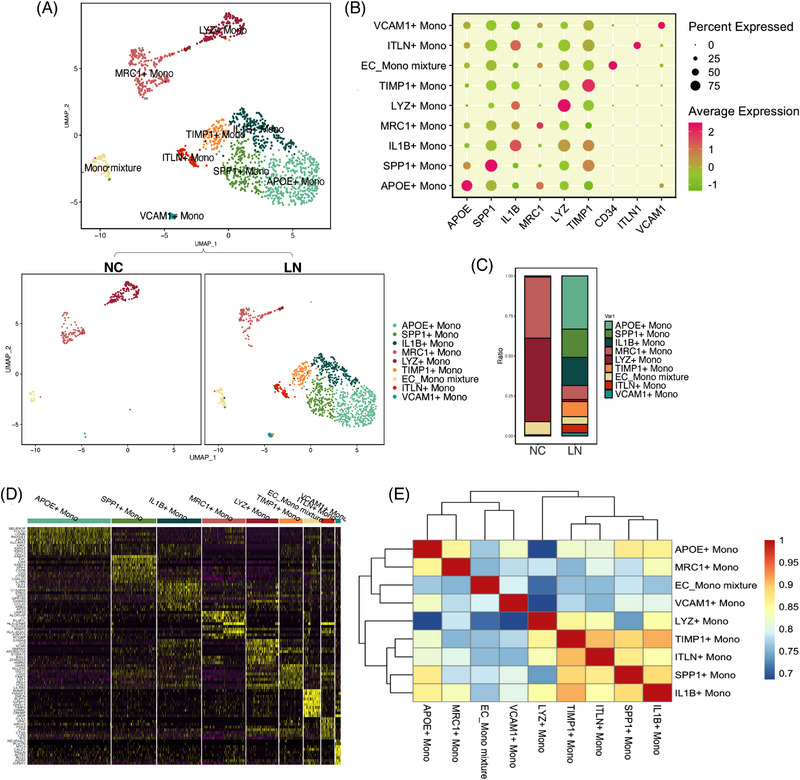
Characterization of mono/MΦ in human normal kidney and lupus nephritis (LN) kidney tissues. (A) Uniform manifold approximation and projection (UMAP) plots showing the composition of mono/MΦ colored by cluster. (B) Dot plots showing average expression of remarkable markers in indicated cell clusters. (C) Ratio of nine major cell types showing in bar plots (in NC group, APOE+ Mono: 0%, SPP1+ Mono: .75%, IL1B+ Mono: 0%, MRC1+ Mono: 38.20%, LYZ+ Mono: 52.06%, TIMP1+: 0%, endothelial cell [EC]_Mono: 8.24%, ITLN+ Mono: 0%, VCAM1+ Mono: .75%; in LN group: APOE+ Mono: 33.22%, SPP1+ Mono: 17.65%, IL1B+ Mono: 17.57%, MRC1+ Mono: 8.83%, LYZ+ Mono: 1.25%, TIMP1+: 9.41%, EC_Mono: 4.83%, ITLN+ Mono: 5.25%, VCAM1+ Mono: 2.00%. (D) Heatmap showing the top ten genes in each cluster of mono/MΦ. (E) The correlation of pairwise mono/MΦ types.

The remodeling of LN patients’ myeloid cells suggested that the cells have unique characters. Nine Mono/MΦ subsets were identified (Figure 5A). Except for the MRC1+ Mono (*n* = 208), EC_Mono mixture (*n* = 80), VCAM1+ Mono (*n* = 26), other Mono/MΦ subclusters were obviously different between normal and LN renal tissues. APOE+ Mono (*n* = 399) were characterized by *APOE*, a type of lipoprotein combined with fats (lipids) in the body to form molecules. Lipoproteins play a crucial role in transporting cholesterol and fats throughout the bloodstream.^42^ In addition, SPP1+ Mono (*n* = 214), IL1B+ Mono (*n* = 211), LYZ+ Mono(n = 154), ITLN+ Mono (*n* = 63) were positive for the relevant markers, respectively (Figure 5B). In LN patients, the proportion of APOE+ Mono, SPP1+ Mono, IL1B+ Mono was dominated (Figure 5C). The APOE+ cells in LN expressed the *SELENOP* (Figure 5D), which may indicate that these cells were surrounded around the blood vessels.^43^ While the expression of *RNASE1* (Figure 5D) could reverse the increased plasma level of extracellular RNA after injury in APOE−/− mice.^44^ Next, we predicted the cell correlation between these Mono/MΦ (Figure 5E).

### 3.6 Mapping of Mono/MΦ subclusters to LN renal tissue

**FIGURE 6:**
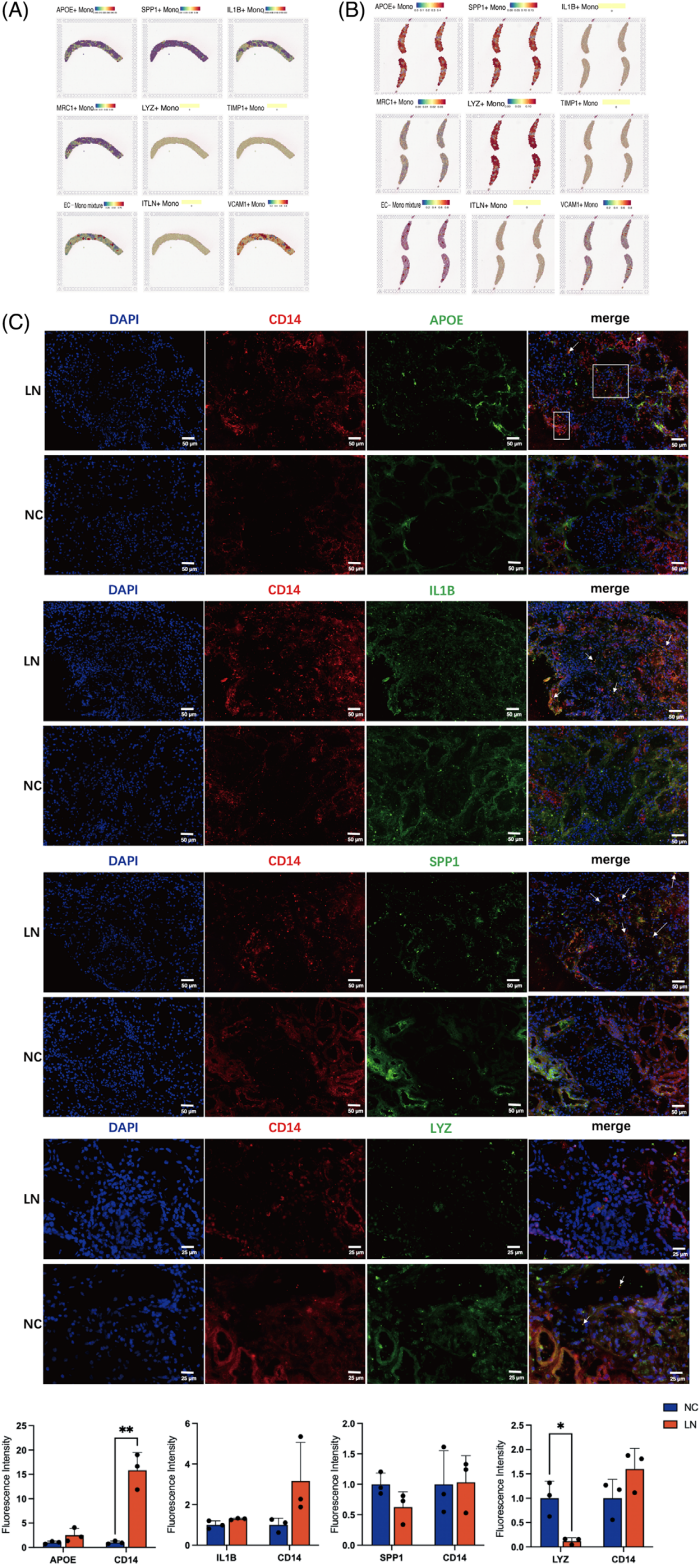
Spatial transcriptome and immunofluorescence staining revealed the existence of lupus nephritis (LN)‐specific mono/MΦ. (A and B) Spatial mapping the APOE+ Mono, SPP1+ Mono, IL1B+ Mono, MRC1+ Mono, LYZ+ Mono, TIMP1+ Mono, endothelial cell (EC)‐Mono mixture, ITLN+ Mono, VCAM+ Mono in tissue sections of LN patients. (C) Immunofluorescence staining of APOE, IL1B, SPP1, LYZ with CD14 in human normal kidney and LN kidney tissues, data are presented as means ± SD and distributions compared by multiple unpaired t test (*p* < .05*; *p* < .01**; *p* < .001***; *p* < .0001****).

To analyze and distinguish the cellular components contained in each 55‐µM diameter spot, we applied the ‘prediction assay’ combined with the single‐cell and spatial transcriptomics. TIMP+ Mono and ITLN+ Mono were not represented in the unsupervised spatial transcriptomic. Since each spot covered multiple cells, the cell type was assigned to the most dominant one in each 55‐µm spot. APOE+ Mono, SPP1+ Mono, and MRC1+ Mono were distributed around the glomerulus and widespread in the renal interstitium (Figure 6A,B). The epithelial cells mixed with Mono/MΦ were mostly localized in the glomerulus, and VCAM1+ Mono were also accumulated in the inner region of the glomerulus, which indicates that the interaction of epithelial cells and Mono/MΦ inside the glomerulus may be involved in LN. The immunofluorescence showed that APOE and CD14 were co‐expressed in the LN patient's kidney, which were not found in the normal ones. Likewise, there were higher co‐expression of IL1B, or SPP1 with CD14 in LN patient‘s kidneys than normal ones, yet the co‐expression of LYZ and CD14 had no significant differences (Figure 6C).

### 3.7 Interaction among myeloid cells contributes to LN

**FIGURE 7:**
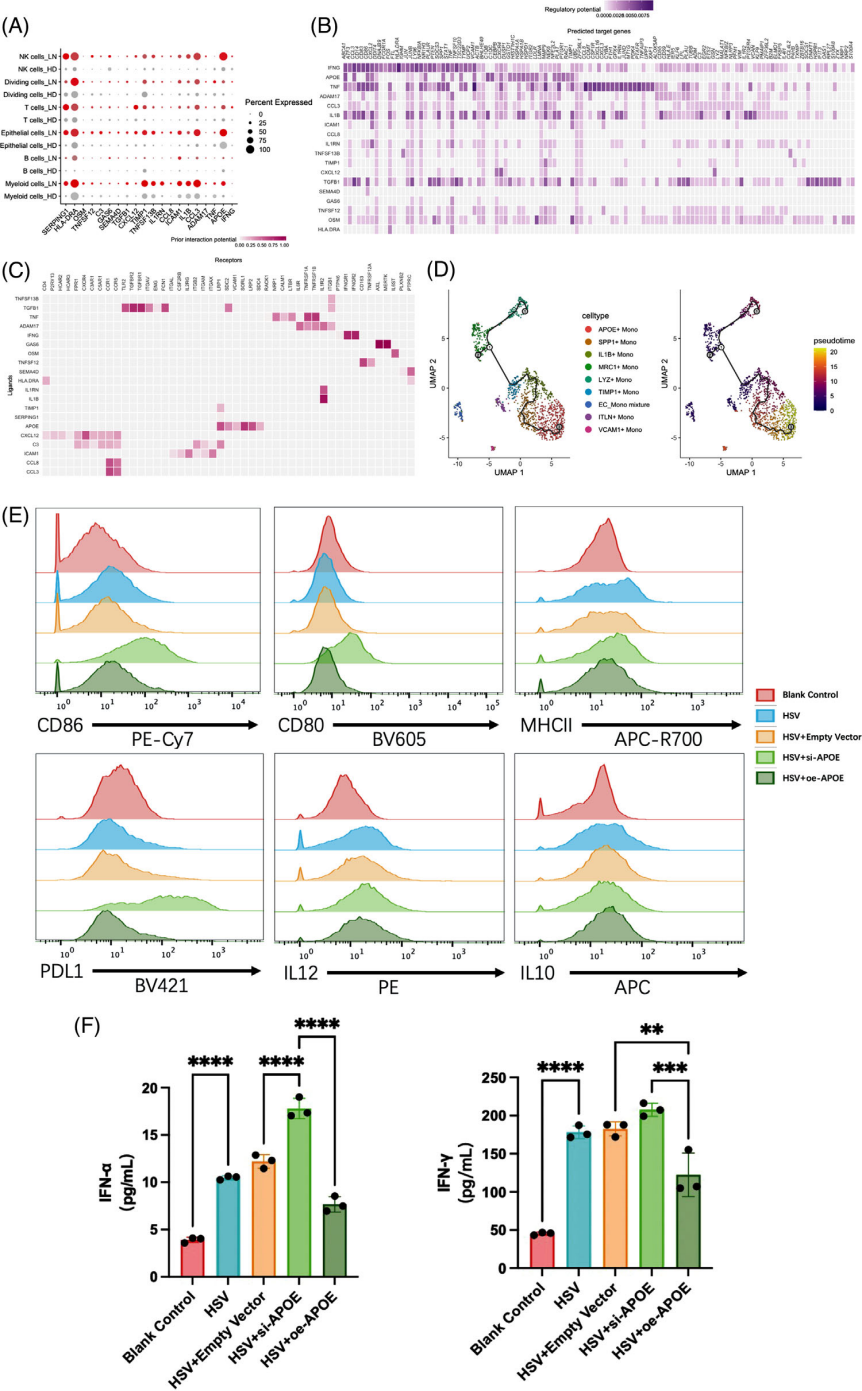
The interaction of immune cells and the function characterize of lupus nephritis (LN)‐specific Mono/MΦ. (A) The top 20 ranked activity ligands of immune cells. (B) Heatmap showing regulatory potential of top 20 ranked ligands and the downstream target genes in immune cells. (C) Ligand‐receptor pairs showing interaction between all immune cells and myeloid cells ordered by ligand activity. (D) Uniform manifold approximation and projection (UMAP) plots showing the developmental trajectory of Mono/MΦ. (E) Flow cytometry evaluated antigen presenting function of RAW264.7 in different groups by staining CD86/80, MHCII, PDL1, IL12, IL10. (F) ELISA was conducted to determine the secretory levels of interferons (IFN)‐α and IFN‐γ, data are presented as means ± SD and distributions compared by Brown‐Forsythe and Welch ANOVA tests (*p* < .05*; *p* < .01**; *p* < .001***; *p* < .0001****).

We used the R package ‘NicheNet’ to examine the interaction between myeloid cells in LN patients. This was achieved by analyzing the expression and interactions between ligands and their corresponding receptors in the cells. Prioritize ligands based on predicted ligand activity, top 20 activity ligands of immune cells were shown in Figure 7A, and the expression of these ligands was significantly higher in LN patients than in normal controls. Through NicheNet analysis, the high prioritized ligands *IFNG*, *APOE*, and *TNF* were the inflammation and immune‐related factors in LN kidneys. The predicted target genes of *IFNG* including *CD14*, *CD83*, *CD86* were the classical markers of myeloid cells, which indicated that the myeloid cells were the main effector cells of IFN‐γ. The *CCL3* and *CXCL12* were the target genes of *APOE*, which may suggest that the chemotaxis of leukocyte was regulated by *APOE* (Figure 7B,C). Furthermore, we conducted correlation analysis of genes and found that APOE was positively correlated with many IFN‐stimulated genes (Figure S4), which further supported the important role of APOE in LN.

### 3.8 Compensatory increase of APOE+ Mono in LN have anti‐inflammatory effect

To clarify the monocyte abnormal development process, the pseudotime analysis by Monocle3 was applied, and it revealed a putative developmental stage of Mono/MΦ differentiation. The results obtained through Monocle3 were consistent with those obtained through Seurat analysis, revealing a similar cell differentiation trajectory. This trajectory displayed the major cell clusters, representing the progression from progenitor to amplifying and then to differentiated cell states. Same as the differences of Mono/MΦ subclusters between LN patients and normal controls (Figure 5A). The MRC1+ Mono, which existed both in LN patients and normal controls, were regarded as the starting point of the differentiation. In normal controls, MRC1+ Mono gradually differentiated to LYZ+ Mono with the function of response to molecule of bacterial origin and positive regulation of cytokine production. However, in the immunological microenvironment of LN, MRC1+ Mono differentiated to APOE+ Mono, in which Mono/MΦ undergo the high expression of *TIMP1*, *IL1B* and *SPP1*, in the temporal gene expression patterns (Figure 7D).To test the function of APOE+ Mono, we assessed CD86, CD80, MHCII, PDL1, IL12, IL10 in RAW264.7 cells which stimulated with HSV and transferred with different plasmids. We found that CD86, CD80, PDL1 increased significantly when the APOE was down‐regulated. The expression of MHCII and IL12 had a tendency of increase, and the IL10 had a tendency of decrease (Figure 7E). The secretory of IFN‐α and IFN‐γ were also assessed with ELISA in different groups, which showed that the IFN‐α and IFN‐γ were elevated with HSV stimulation compared to the blank control. Moreover, the down‐regulated of APOE raised the secretory of IFN‐α and IFN‐γ while the up‐regulated of APOE reversed this outcome (Figure 7F). Taken together, APOE+ Mono represented the differentiation endpoints of monocytes under the immune microenvironment of LN. APOE may be the anti‐inflammatory factor in LN, and the decreased antigen presenting function of APOE+ Mono can be regarded as the self‐rescue of body.

### 3.9 Lymphangiogenesis contributes to Mono/MΦ’s trafficking to glomeruli

**FIGURE 8:**
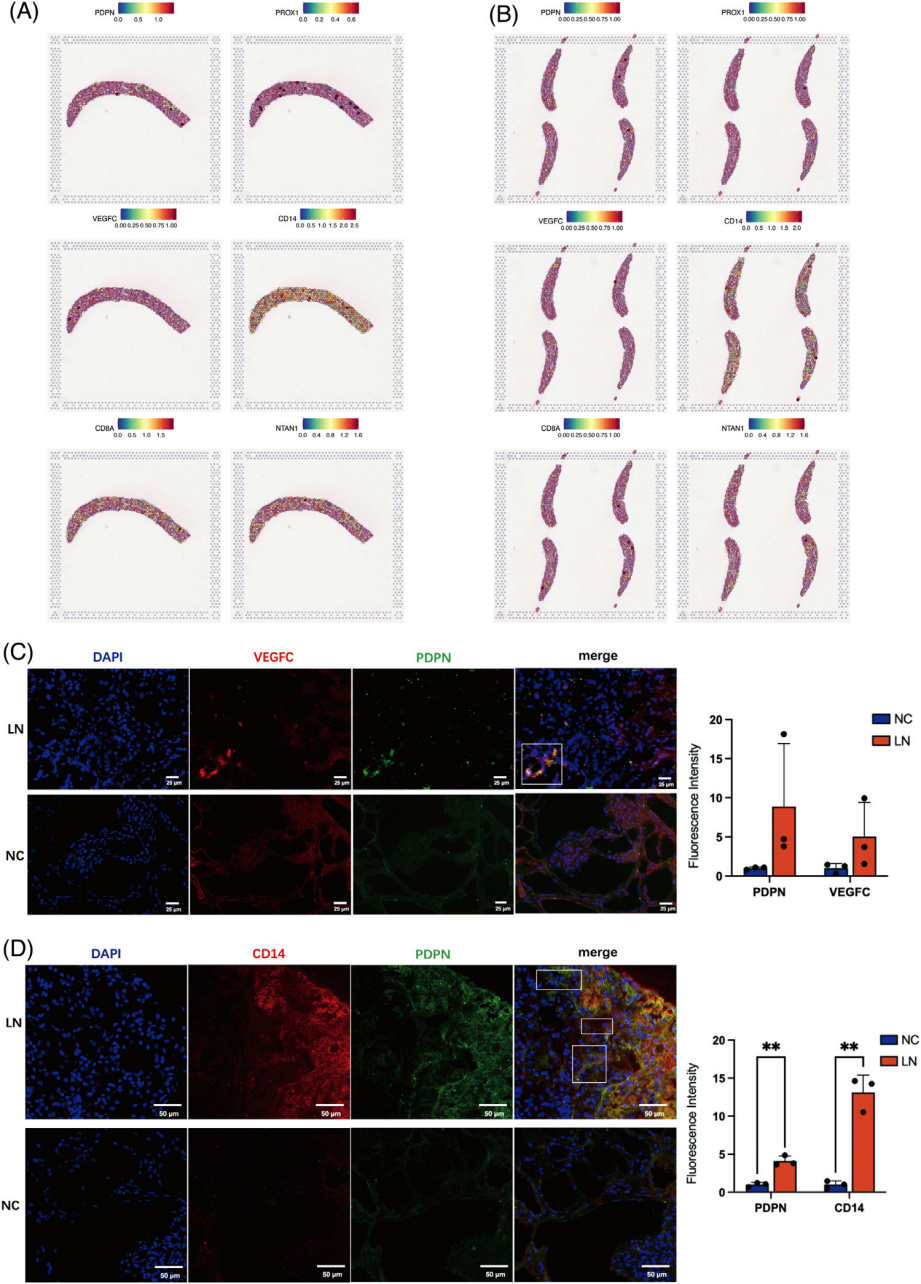
The lymphangiogenesis existence in lupus nephritis (LN) patients. (A and B) Spatial feature plots of gene expression of PDPN, PROX1, VEGFC, CD14, CD8A, NTAN1 in kidney tissue sections of LN patients. (C) Double immunofluorescence staining of VEGFC and PDPN in human normal kidney and LN kidney tissues. (D) Double immunofluorescence staining of CD14 and PDPN in human normal kidney and LN kidney tissues, data are presented as means ± SD and distributions compared by multiple unpaired t test (*p* < .05*; *p* < .01**; *p* < .001***; *p* < .0001****).

After identifying LN monocyte subsets and their locations, it is necessary to elucidate how these clusters were accurately transported in inflammatory loci. As reported, immune cells in various renal diseases could be transported by lymphatic vessels and tertiary lymphatic system to kidney. We mapped the lymphangiogenesis and lymph node‐related markers in spatial transcriptomic slices. *PDPN* was continuously expressed in a cord‐like structure along the distribution of glomerulus. The expression of *PROX1* and vascular endothelial growth factor C (*VEGFC*) was found to be closely linked to *PDPN*. The expression of *CD14* was found to be highly correlated with the distribution of markers for lymphangiogenesis (Figure 8A,B), reflecting that CD14+ Mono/MΦ enter the vicinity of the glomerulus through newly forming lymphatics in LN kidney. Consistent with the above results, immunofluorescence co‐staining certificated that PDPN and VEGFC were distributed at the same area in LN kidney (Figure 8C), which reflected the lymphangiogenesis in LN kidneys. Meanwhile, the expression of CD14 and PDPN in LN patients was shown as the double contour, which suggested that the CD14+ Mono/MΦ were infiltrated beside the lymphatics (Figure 8D).

## 4 DISCUSSION

LN is characterized by an inflammatory response with significant immune system dysfunction. In order to explore the underlying inflammatory network, it is necessary to target abnormal immune cell clusters which play roles in triggering this local immune response. Here, we used bioinformatic techniques to reconcile single‐cell and spatial transcriptomic data and truly discovered some interesting clues verified by immunofluorescence and cell experiment.

Interestingly, under our analysis, myeloid cell is one special cell group with unique character in LN renal tissue. Since the LYZ+ Mono only existed in normal controls, we could observe the whole LN renal abnormal monocyte development process from LYZ+ Mono to TIMP+ Mono, SPP1+ Mono, IL1B+ Mono, and eventually APOE+ Mono by pseudotime analysis. APOE+ Mono were shown in the endpoint of pseudotime analysis possessing the decreased function of antigen presentation and the lower secretory of IFN‐α and IFN‐γ, which suggests that this Mono/MΦ cluster may be a kind of beneficial subsets of LN. The primary producers of APOE in local tissues are the liver and macrophages, and it plays a role in regulating cholesterol metabolism. The levels of APOE in the blood of people with SLE have been found to have a positive association with their SLEDAI scores, the presence of anti‐dsDNA antibodies and cytokines such as IL‐6, IFN‐γ and IL‐10. However, there was no significant correlation with the amounts of total cholesterol and triglycerides, as compared to those in healthy individuals.^45^ APOE facilitates the recognition and presentation of lipid antigens in serum by APCs. It acts as a mediator by capturing antigens, including lipids from infected cells, and presenting them to nearby APCs.^46^ Most research of atherosclerosis showed that the deficiency of APOE would enhance the inflammation and the formation of plaque,^47^, ^48^ which is consistent with our findings that the APOE+ Mono had the function of antigen presentation. APOE in various pathological processes has different effects either pro‐inflammation or anti‐inflammation. APOE increased the levels of Th1 cytokines in the serum and decreased the production of IL‐4 in sepsis, which is the main Th2 cytokine produced by Natural killer T cells.^49^ The lack of APOE in mice made them more vulnerable to *K. pneumoniae* infection, causing a reduction in the ability of granulocytes to phagocytize and resulting in greater bacterial growth and increased death.^50^ In cancer, APOE has been demonstrated to trigger strong anti‐tumour effects and amplify T cell activation during different immune‐based treatments.^51^ Different to the above results, most research of atherosclerosis showed that the deficiency of APOE would enhance the inflammation and the formation of plaque,^47^, ^48^ which means the APOE protein is anti‐inflammatory. Previous research has indicated that the levels of APOE in the peripheral blood and renal tissue of individuals with lupus were elevated compared to healthy individuals and were linked to the severity of the disease. Additionally, the mRNA expression of APOE in monocytes of lupus patients was found to be elevated. Researchers speculated that this may be a compensatory response of the body to elevate APOE in order to suppress excessive Th1‐type immune response and inflammation.^52^, ^53^ Our LN research preliminarily reveals that the up‐regulated of APOE decreases the antigen‐presenting capacity of monocytes in LN, it still needs deeper and further exploration and verification.

During the monocyte cluster differentiation process, *TIMP1, IL1B, and SPP1* take turns in mediating the disease process. TIMP1+ Mono were at the early stage of the monocyte differentiation. The mRNA expression of *TIMP1* which was correlated with SLEDAI score^54^ may contribute to the pathogenesis of LN.^55^ The expression of the *IL1B* was found to be significantly elevated after stimulation with lipopolysaccharide (LPS) and adenosine triphosphate (ATP) in peripheral monocytes of SLE patients,^56^ indicating that these IL1B+ monocytes were activated by the inflammatory stimulus in LN. SPP1, a pleiotropic protein, is important in bone remodeling and immune system signaling. Increased expression of *SPP1* has been linked to the development of autoimmune diseases (i.e., SLE).^57^ Taken together, part of LN‐specific subclusters enhanced the auto‐immunoreaction and inflammation in the kidney.

In DC subgroups, CD1C+CLEC10A+DC was a new cluster found by recent studies.^58^, ^59^, ^60^ The activation of CD1C+ DCs by CLEC10A ligands resulted in an increase in IL‐8, IL‐10^58^ and TNF‐α levels in response to toll‐like recptor (TLR) stimulation. Recent evidence has shown that CLEC10A secretion can be incorporated into compartments rich in human leukocyte antigen (HLA). I and II in human immature DCs derived from monocytes,^61^ indicating that the presence of CLEC10A on CD1C+ DC boosts both pro‐inflammation and antigen presentation.

Another worthy exploration is their accurate transport to renal environment since immune cell infiltration is a vital pro‐inflammatory mechanism in SLE.^62^ As lymphogenesis markers, *PDPN* and *VEGFC* were stably expressed along with the distribution of glomerulus, which represented that the lymphangiogenesis was connected with local inflammation distributed around the lesion and indicated that under the assistance of lymphogenesis, para‐glomerular immune center formed. The TGFβ‐VEGFC pathway stimulates the production of VEGFC in tubular epithelial cells, macrophages and mesothelial cells, resulting in lymphangiogenesis in renal and peritoneal fibrosis.^63^ With the expression of *CD14* and *PDPN* in LN kidneys (Figure 8D) and the function enrichment of Mono/MΦ (Figure 8E), we inferred that the CD14+Mono transport to the lesion of auto‐immune and inflammation center to present the autoantigen by the neo‐lymphatic induced by the immune microenvironment of LN.

In this study, we combined single‐cell and spatial transcriptomics to map all major LN‐associated cell types and spatially located them to the different regions. Although the scRNA‐seq of kidney and blood of LN patients had been studied, we firstly mapped the LN‐specific mono/MΦ subclusters in the spatially transcriptomic slices and found the lymphangiogenesis in LN which may be the channel of the LN‐specific mono/MΦ to entry and exit the glomerulus lesion. Our study has provided insights into the immune landscape and the specific Mono/MΦ function in the microenvironment of LN. This might allow the possibility of developing therapeutic approaches that target APOE+ Mono to inhibit the initiation of auto‐immune responses without influence the downstream T and B cells. Further studies are needed to identify the mechanisms by which APOE+ Mono regulates lymphocyte infiltration in LN kidney tissue, as a critical step in reversing the overactive autoimmune responses seen in LN. We supposed to develop small‐molecule agonists targeting APOE, which could weaken the antigen presentation of LN pathological monocytes, thereby suppressing spontaneous immune responses. However, the above schedule should be based on detailed experiment basis which we are on our way.

## 5 CONCLUSION

In summary, by scRNA‐seq and spatial transcriptome, we found that APOE+ Mono was a specific subset of monocytes in LN, and with the up‐regulated of APOE in monocytes, the capacity for presenting antigens and secretion of IFN were significantly reduced. The lymphangiogenesis in LN prompted the trafficking of Mono/MΦ in LN kidney. Taken together, our multi‐omics approach further clarifies the pathogenesis of LN and identifies potential therapeutic targets.

## CONFLICT OF INTEREST STATEMENT

The authors declare no competing interests.

## Supporting information

Supporting Information
