# Supplementary material for: Immune landscape and the key role of APOE+ monocytes of lupus nephritis under the single‐cell and spatial transcriptional vista
Source: Clin Transl Med. 2023 Apr 7;13(4):e1237. doi: 10.1002/ctm2.1237 (PMC10080215; doi:10.1002/ctm2.1237)
Supplement: Supplementary file 1 — Supporting Information [file CTM2-13-e1237-s001.docx]

Supplementary figures and table


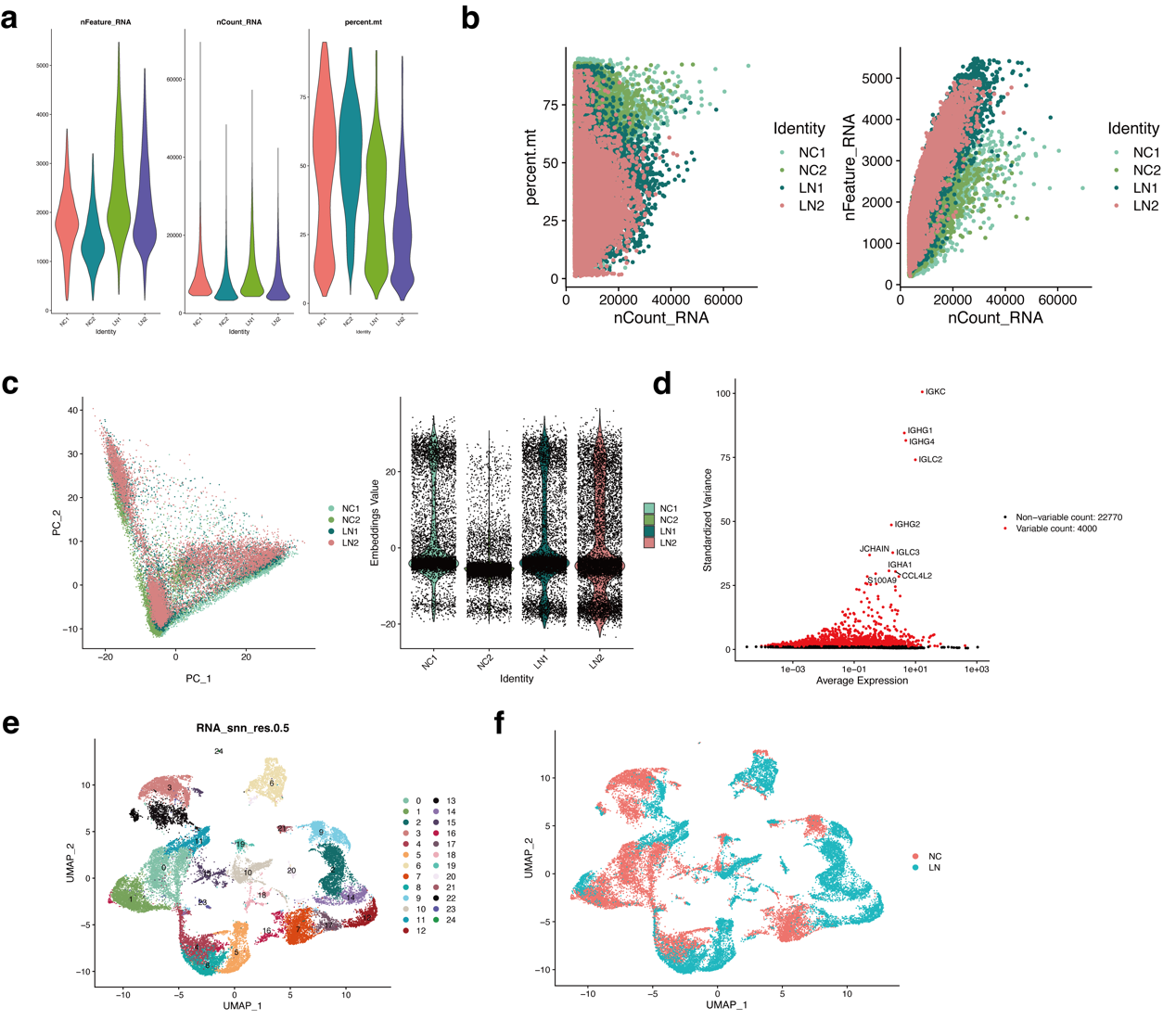


**Supplementary figure 1.** The quality control of scRNA-seq. a. The numbers of UMI counts, gene features, and percentages of mitochondrial genes after quality control in 4 samples. b. The feature scatter of nCount_RNA, nFeature_RNA and percent.mt. c. The integration degree of two dimensions in 4 samples. d. The high variable genes in the samples. e. UMAP plot showing clusters of cells in the resolution of 0.5. d. UMAP plot showing the overall view of cells in LN and NC group.


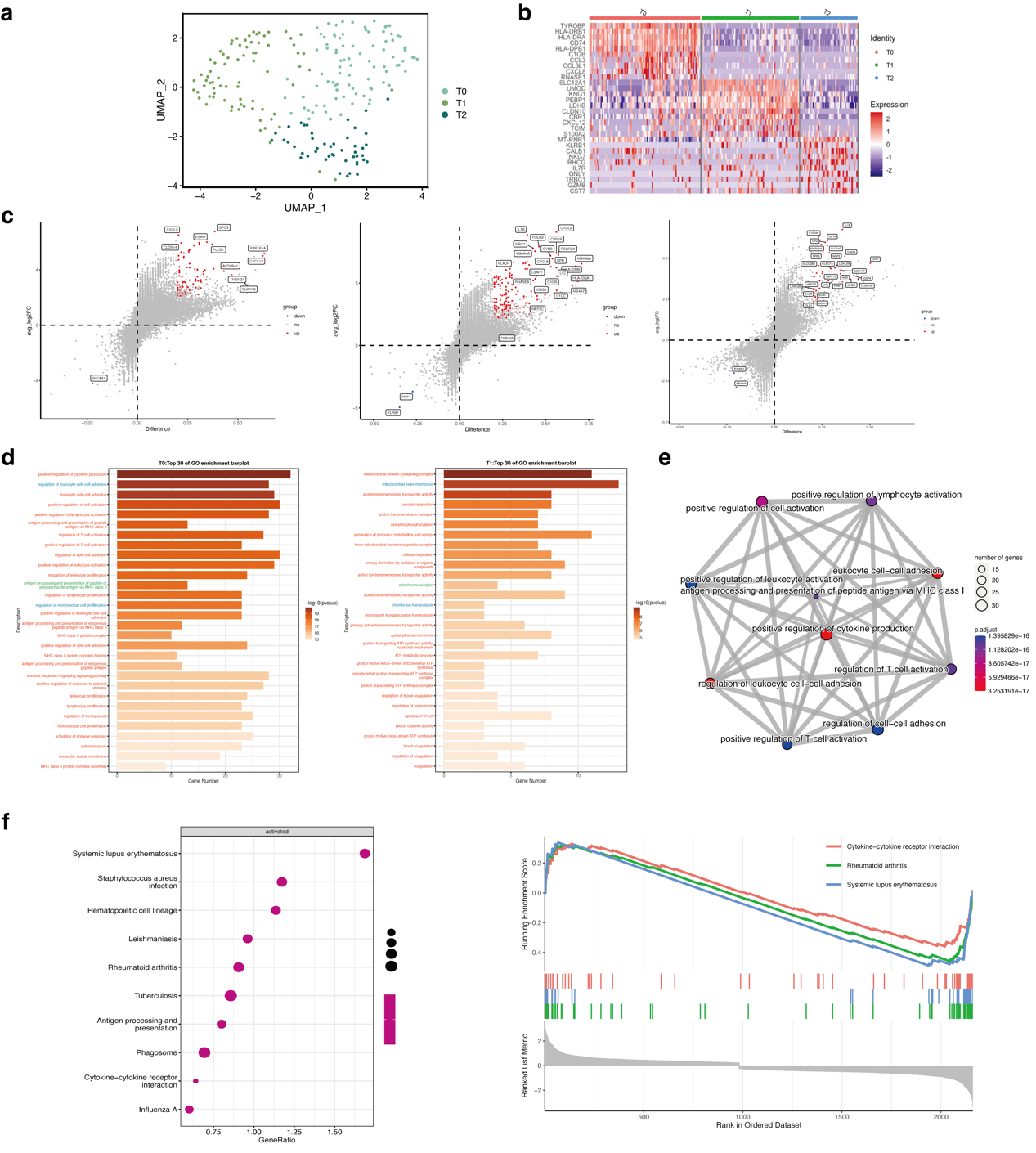


**Supplementary figure 2.** Characterization of T cells in human normal kidney and LN kidney tissues. a. UMAP plots showing the composition of T cells colored by cluster. b. Heatmap showing the top ten genes in each cluster of T cells. c. Volcano plots showing the high expression genes in T1 versus T2 (left), T0 versus T2 (middle), T0 versus T1(right). d. GO enrichment of T0 (left) and T1(right) high expression pathway. e. Emapplot of pathway relationship in T0 cluster. f. GSEA Enrichment showing the active pathways of T cells.


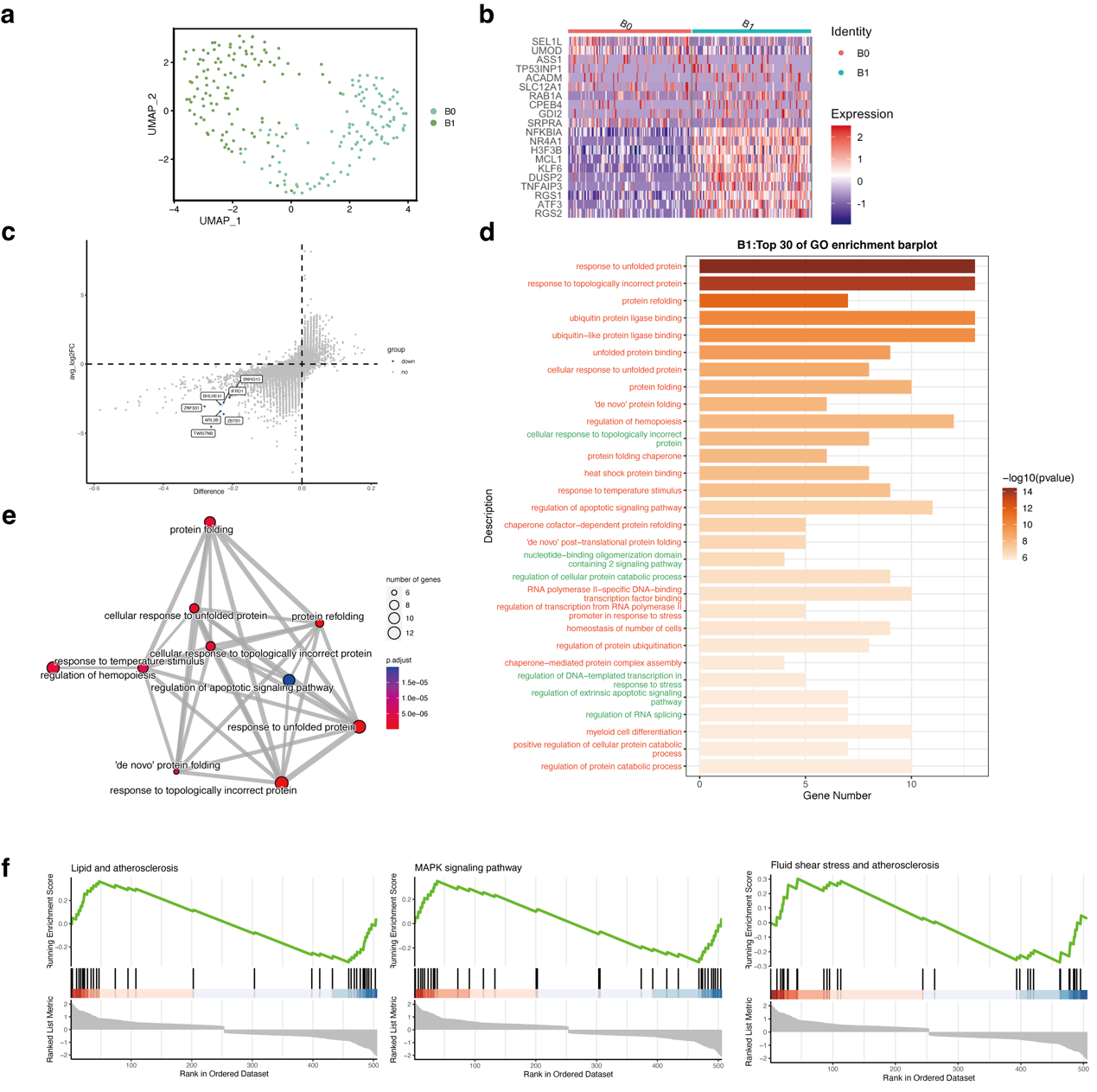


**Supplementary figure 3.** Characterization of B cells in human normal kidney and LN kidney tissues. a. UMAP plots showing the composition of B cells colored by cluster. b. Heatmap showing the top ten genes in each cluster of B cells. c. Volcano plots showing the high expression genes in B0 versus B1. d. GO enrichment of B1 high expression pathway. e. Emapplot of pathway relationship in B1 cluster. f. GSEA Enrichment showing the active pathways of B cells.


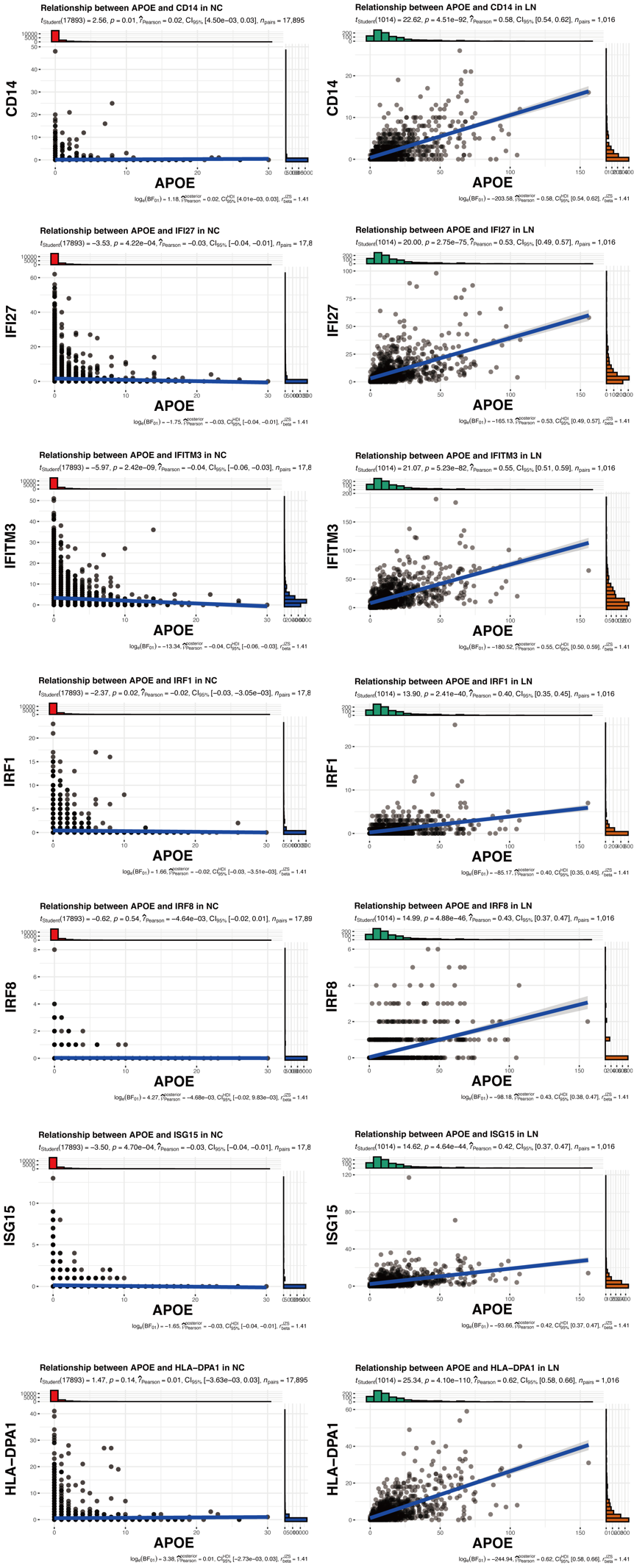


**Supplementary figure 4.** The gene correlation between APOE and other IFN-related genes(IFI27, IFITM3, IRF3, IRF8, ISG15) and antigen presenting related genes(CD14, HLA-DRA1) in human normal kidney and LN kidney tissues.

**Supplementary Table 1**. The information of LN patients.

| Patient | Age | scRNA-seq | Spatial transcriptome | Sex | stage of LN |
| --- | --- | --- | --- | --- | --- |
| T1 | 15 | yes | no | Female | IV |
| T2 | 29 | yes | no | Female | IV |
| T3 | 33 | no | yes | Female | IV |
| T4 | 23 | no | yes | Female | IV+V |
